# Concepts and Misconceptions Concerning the Influence of Divalent Ions on the Performance of Reverse Electrodialysis Using Natural Waters

**DOI:** 10.3390/membranes13010069

**Published:** 2023-01-05

**Authors:** Joost Veerman

**Affiliations:** REDstack bv, 8606 BT Sneek, The Netherlands; j.veerman@redstack.nl

**Keywords:** salinity gradient power, salinity gradient energy, renewable energy, blue energy, multivalent ions, divalent ions, ion exchange membranes, membrane resistance

## Abstract

Divalent ions have a negative effect on the obtained power and efficiency of the reverse electrodialysis (RED) process when using natural waters. These effects can largely be attributed to the interaction between the various ions and the membranes, resulting in a decreased membrane voltage, an increased membrane resistance, and uphill transport of divalent ions. The aim of this study was to investigate the causes of these differences and, if possible, to find underlying causes. The approach mainly followed that in literature articles that specifically focused on the effect of divalent ions on RED. It transpired that seven publications were useful because the methodology was well described and sufficient data was published. I found two widely shared misconceptions. The first concerns the role of the stack voltage in uphill transport of divalent ions; itis often thought that the open circuit voltage (OCV) must be taken into account, but it is plausible that the voltage under working conditions is the critical factor. The second debatable point concerns the methodology used to make a series of solutions to study the effect of divalent ions. Typically, solutions with a constant number of moles of salt are used; however, it is better to make a series with a constant ratio of equivalents of those salts. Moreover, it is plausible that the decreased voltage can be explained by the inherently lower Donnan potential of multi-charged ions and that increased resistance is caused by the fact that divalent ions—with a lower mobility there than the monovalent ions—occupy relatively much of the available space in the gel phase of the membrane. While both resistance and voltage play a decisive role in RED and probably also in other membrane processes like electrodialysis (ED), it is remarkable that there are so few publications that focus on measurements on individual membranes. The implications of these results is that research on the effect of divalent ions in RED, ED and similar processes needs to be more structured in the future. Relatively simple procedures can be developed for the determination of membrane resistance in solutions of mixtures of mono- and divalent salts. The same applies to determining the membrane potential. The challenge is to arrive at a standard method for equipment, methodology, and the composition of the test solutions.

## 1. Introduction

It was Richard Pattle who in 1954 first proposed to use salinity gradients for generation of electrical energy, a method now known as reverse electrodialysis (RED) [1]. The main potential of salinity gradient energy (SGE) is the combination of seawater and river water, also known as Blue Energy [2]. These natural sources are rich in a wide range of ions and the effects of these ions on the produced power are the subject of this study. In laboratory experiments using pure NaCl solutions—with salinities comparable to river and sea water—power densities are achieved of more than 2 W/m^2^ [3]. However, with natural feed waters, there is the problem of biological matter, solid particles, and dissolved matter as sources of membrane fouling and spacer blockage, resulting in a reduction in power density [4,5,6,7,8]. Possible threats to the anion exchange membranes (AEM) and the cation exchange membranes (CEM) from divalent ions were mentioned as early as 1970 by Lacey in an article citing the Great Salt Lake in Utah as a possible source of RED, along with freshwater from one of the incoming rivers [9]. In addition to sodium and magnesium ions, this water also contains iron and manganese ions that can be detrimental to membranes. Moreover, even with pure NaCl solutions, the open circuit voltage and the power density decrease significantly when divalent ions are added [10,11,12,13,14,15,16,17,18,19] and these effects are the topic of this paper.

The concentrations of ions in the open sea are more or less the same everywhere in the world, but in rivers and estuaries they can differ considerably. Table 1 shows the composition in Lake IJssel (fed by the river IJssel) and in the Wadden Sea (in open connection with the North Sea) in the Netherlands. These two bodies of water are separated by the Afsluitdijk (closure dam). This where the pilot plant of the company REDstack b.v. is located and the RED technology is being developed for commercial applications [20]. The concentrations in Table 1 were used in the following considerations on the effect of multivalent ions in RED.

In order to make the effects of divalent ions manageable for RED technology, two methods have usually been followed: removing these ions by pretreatment of the feed waters [23] and modification of the membrane [17]. In fact, there are three options for the latter. The first uses electrostatic repulsion; an example is the Neosepta CMX where a cationic polyelectrolyte layer (that can be considered as a thin AEM) repels the double charged magnesium and calcium ions more than the single charged sodium ions [10]. The second method uses steric exclusion by applying a high cross-linked coating on the membrane. The hydrated divalent sulfate ion is larger than the hydrated chloride ion and is less likely to pass through this layer. The Neosepta ACS membrane (an AEM) owes its special properties to this effect [10]. Sometimes, combinations of both methods are applied, or the membrane is built by layer-by-layer coatings [24]. The application of monovalent selective membranes at RED initially seemed successful [10,12]. However, the resistance of these membranes is higher than that of the normal types, and using these membranes results in not the whole thermodynamic potential of the salinity gradient power being used. Moreover, there are no results described in the scientific literature of using these membranes for prolonged times. One scenario is that the divalent ions diffuse slowly into the monovalent membranes, thereby negating the protecting effect [25]. Therefore, a third option was developed: applying very open membranes [26]. Examples are the Fujifilm Type I membranes (CEM and AEM). Due to the open structure, a high permeability for monovalent and divalent ions is combined with a low resistance. Unfortunately, the consequence of this is that the permselectivity for counter ions is also low [27].

Before effective countermeasures can be taken, knowledge is needed regarding the origin of the power decreasing effect of divalent ions. The literature describes many dozen causes of the effect of multivalent ions on RED power. These can be summarized as follows: (i) decreased membrane voltage and permselectivity, (ii) increased membrane resistance, (iii) uphill transport of multivalent ions, and (iv) formation of ion pairs with the fixed charges. These effects were investigated using available data in the relevant literature and are discussed in the next section. These data are sometimes tables, but also were often graphs that were digitized for this work.

## 2. Fundamentals

I will start with the theory of a pure solution of a single salt. The voltage *E* over a cation exchange membrane caused by a salinity gradient is given by the Donnan equation:

(1) $$E=\alpha_{CEM}\frac{RT}{zF}\ln\left(\frac{a_{H}}{a_{L}}\right)=\alpha_{CEM}\frac{RT}{zF}\ln\left(\frac{\gamma_{H}C_{H}}{\gamma_{L}C_{L}}\right)$$

where *α_CEM_* stands for the permselectivity of the membrane, *R* for the gas constant (8.314 J mol^−1^K^−1^), *T* the temperature (K), *z* the valence of the ion and *F* the Faraday constant (96_,_485 C mol^−1^); activities are denoted by *a*, activity coefficients by *γ* and concentrations (in mol/L) by *C*. Subscripts *H* and *L* are used for High and Low concentration solutions. Activity coefficients in the lower concentration range can be obtained with the extended Debye–Hückel equation:

(2) $$\log\left(\gamma \right)=\frac{-0.51z^{2}\sqrt{I}}{1+\frac{A}{305}\sqrt{I}}\;with\;I=\frac{1}{2}\underset{i}{\sum }z_{i}^{2}C_{i}$$

where *A* stands for the diameter of the hydrated ion (in pm) and *I* for the ionic strength of the solution. Some values for *A* are: Na^+^: 450 pm, Cl^−^: 300 pm, Mg^2+^: 800 pm, Ca^2+^: 600 pm, and SO_4_^2−^: 400 pm [28]. For an ideal stack the generated electrical power (P_ideal_) can be written [29] as:

(3) $$P_{ideal}=\frac{N^{2}E^{2}}{4R_{i}}$$

where *N* stands for the number of cell pairs, *E* for the EMF (electromotive force) of one cell pair (V) and *R_i_* for the resistance of that cell pair (Ω). With constant *N*, maximum power is achieved with maximum *E* and minimum *R_i_*.

## 3. Effects of Multivalent Ions on RED Power

### 3.1. Uphill Transport

**Figure 1:**
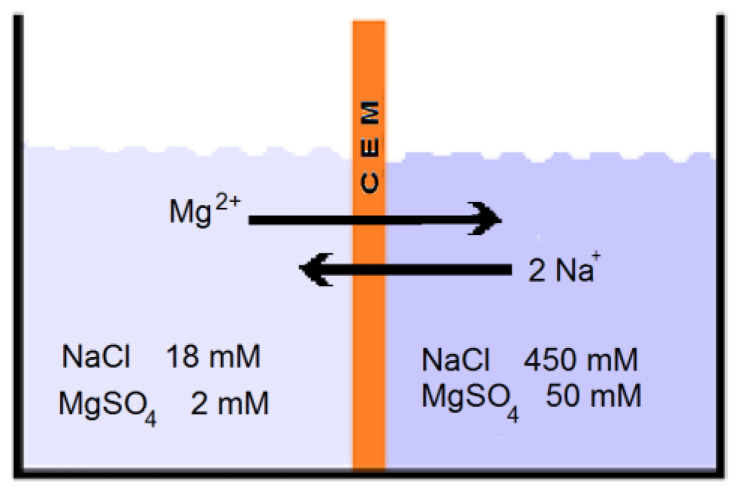
Principle of uphill transport of magnesium ions.

Figure 1 shows a simple experiment: two vessels with NaCl/MgSO_4_ solutions are separated by a CEM. Assuming an ideal membrane (*α_CEM_* = 1) and ionic activity coefficients of unity, the membrane voltage due to the sodium ions can be calculated using Equation (1) (82 mV), as can the voltage due to magnesium ions (41 mV). The measured voltage is somewhere between these values and in any case higher than the magnesium voltage. This results in transport of magnesium ions from the low to high concentration compartment, a process called uphill transport. Meanwhile, to obey the law of electroneutrality, sodium ions move from high to low concentration. The process ends if equilibrium is established and both voltages are equal.

The concept of uphill transport by IEMs has been known for much longer. Castilla et al. [30] published a theoretical study on uphill transport in RED stack for two cases: control of the current through the system and control of the electrical potential. Moya wrote a theoretical paper concerning the effect of uphill transport in the RED process and argued the usefulness of removing divalent ions [31].

The situation as depicted in Figure 1 is analogous to an open (not connected to a load) RED stack that is fed with solutions that contain equal percentages of MgSO_4_. Under zero current conditions, no net charge is transferred through each membrane and the back transport of each divalent ion is compensated through the transport of two monovalent ions. Many publications use this reasoning of open stacks to explain the negative effect of divalent ions on the generated power of RED stacks [3,14,15,27,32]. However, this approach is not very realistic because, under power generating conditions, the voltage across the stack is approximately half the OVC and the same applies to each individual membrane. An exception is the publication by Post et al. where uphill transport (i.e., back transport against the salinity gradient) was studied under current-producing conditions [10]. Unfortunately, the current in their experiments was rather low (10 A/m^2^) and probably lower than needed for maximum power delivery.

**Figure 2:**
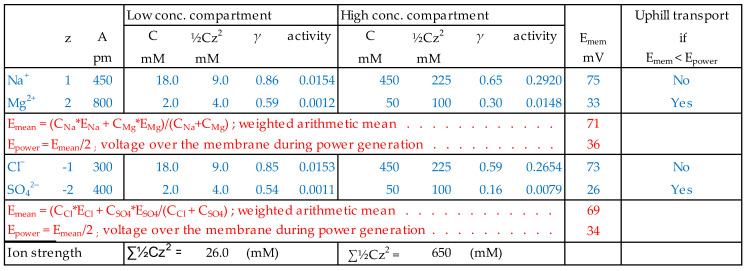
Decision scheme for uphill transport in the case of equal relative composition of High and Low feed waters.

For the RED experiment with equal MgSO_4_/NaCl ratios in the high and low concentration compartments (HC and LC), three cases can be distinguished regarding the load on the stack. With an open stack, there will certainly be uphill transport of divalent ions, and with a short-circuited stack, certainly not. In the case of a stack that is loaded to deliver maximum power, uphill transport is at first sight unlikely if concentrations instead of activities are used in the calculation of membrane potentials. In Figure 2, sophisticated calculations are made; activity coefficients were generated here by using Equation (2).

**Figure 3:**
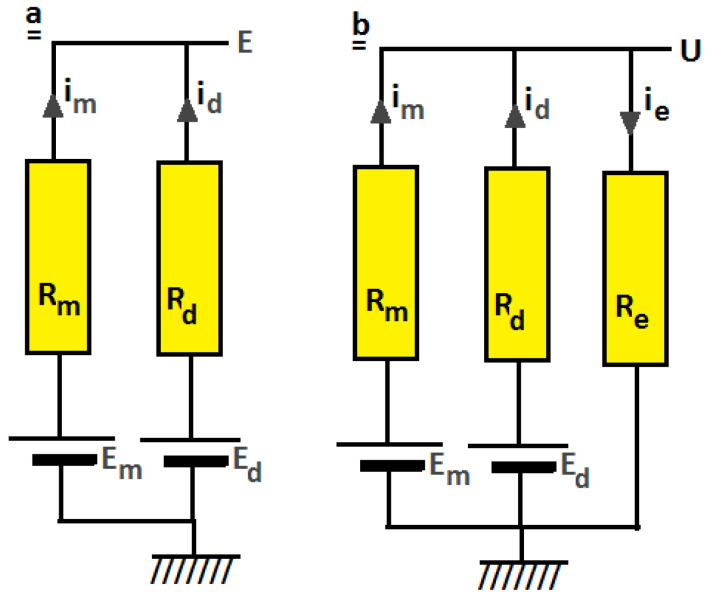
Model of a RED-stack operating on a mixture of mono- and divalent ions without an external resistance (**a**) and with an external resistance (**b**).

Membrane potentials were estimated as the weighted mean of monovalent and divalent potential.

(4) $$E_{mem}=x_{mono}\cdot E_{mono}+x_{di}\cdot E_{di}$$

where *x_mono_* and *x_di_* are the mol fractions of the mono- and divalent ions and *E_mono_* and *E_di_* are the membrane potentials generated by these ions. This linear relationship between the mol fraction and the OCV is a rough interpretation of the findings of Vermaas et al. in Figure 3 of their publication about the influence of multivalent ions on RED power [12]. The conclusion is that there is an uphill transport of magnesium ions through the CEM because the membrane potential under power delivering condition is 36 mV, whereas the magnesium part of the potential is a little lower (33 mV). The net driving voltage for the magnesium ions is only 3 mV and the magnesium flow is expected to be very low. Something similar applies to the transport of sulfate ions through the AEM, although in this case, the net driving force is a little higher (8 mV). As seen in Table 1, the concentrations of divalent ions in Lake IJssel are much higher than in the sea, and in this case, uphill transport of both divalent anions and cations will occur.

Conclusion. If the fraction of the divalent ions in the LC stream is higher than in the HC stream, uphill transport is a fact during power delivery. On the other hand, if this fraction is significant lower, then uphill transport is unlikely.

### 3.2. Stack Voltage

Vermaas et al. proposed a RED model for mixtures of mono- and divalent ions [12]. It consisted of two parallel circuits for the action of monovalent and divalent ions over an ion exchange membrane (Figure 3a). With this model, uphill transport can be explained if there is no external current (i.e., open circuit condition). However, the situation is different under power generating conditions. Therefore, I added an external load to this model to calculate the delivered power and internal currents under working circumstances. Figure 3b shows this extended model for one membrane. Variables *E_m_* and *E_d_* are the electromotive forces of the mono- and divalent systems, *R_m_* and *R_d_* the internal resistance of the membrane specified for each ion, and *R_e_* is the external applied resistance. Electrical currents are *i_m_*, *i_d_*, and *i_e_*. For considerations about uphill transport of divalent ions, the terminal voltage *U* is important.

The system of Figure 3b is described by four equations:

(5) $$E_{m}-U=i_{m}\cdot R_{m}$$

(6) $$E_{d}-U=i_{d}\cdot R_{d}$$

(7) $$U=\left(i_{m}+i_{d}\right)\cdot R_{e}$$

(8) $$i_{e}=i_{m}+i_{d}$$

These four equations contain four unknowns: *U*, *i_m_, i_d_* and *i_e_*. The solution for *U* is:

(9) $$U=R_{e}\frac{E_{m}R_{d}+E_{d}R_{m}}{R_{e}R_{d}+R_{e}R_{m}+R_{m}R_{d}}\;\;$$

With the solution of *U* the three other unknowns are now known:

(10) $$i_{m}=\frac{E_{m}-U}{R_{m}}\;\;\left(a\right);\;\;\;\;\;i_{d}=\frac{E_{d}-U}{R_{d}}\;\;\left(b\right);\;\;\;i_{e}=i_{m}+i_{d}\;\;\;\;\;\left(c\right)\;\;\;\;$$

The delivered power to the load is then:

(11) $$P=U\cdot i_{e}$$

Variables *E_m_* and *E_d_* can be calculated from the ionic composition on both sides of the membrane using Equations (1) and (2). The value of *U* is now dependent on *R_m_*, *R_d,_* and *R_e_*. However, if *R_e_* is adjusted for maximum power dissipation in *R_e_*, the resulting value of *U* is only dependent on *R_m_* and *R_d_*. Maximum power is achieved for d*P*/d*R_e_* = 0. It follows that for the external resistance at maximum power (*R_e,max_*):

(12) $$\frac{1}{R_{e,max}}=\frac{1}{R_{m}}+\frac{1}{R_{d}}$$

The maximum power (*P_max_*) is then:

(13) $$P_{max}=\frac{{\left(E_{m}R_{d}+E_{d}R_{m}\right)}^{2}}{4R_{m}R_{d}\left(R_{m}+R_{d}\right)}$$

Or in terms of conductivity G:

(14) $$P_{max}=\frac{1}{4}\frac{{\left(E_{m}G_{m}+E_{d}G_{d}\right)}^{2}}{\left(G_{m}+G_{d}\right)}\;\;with\;\;\;G_{m}=\frac{1}{R_{m}}\;and\;\;\;\;G_{d}=\frac{1}{R_{d}}$$

Conclusion. Equation (14) gives an expression of the maximum power generated by an IEM in a mixture of mono- and divalent ions in terms of membrane voltages and ionic conductivities. The conductivities *G_m_* and *G_d_* are dependent on the mobilities and concentrations of the ions in the gel phase of the membrane, whereas the membrane voltages *E_m_* and *E_d_* follow from Equations (1) and (2).

### 3.3. Effect of Divalent Ions on RED Power

From Table 2 it follows that substitution of one equivalent Na^+^ by one equivalent Mg^2+^ or Ca^2+^ has no increasing effect on the resistance within the feed water compartments but has a small decreasing effect. Consequently, negative effects on power production in RED can mainly be attributed to interaction of those divalent ions with the membranes. Given Equation (3), it is logical to determine the effect of divalent ions on R_i_ and E of the individual membranes (AEM and CEM).

Resistance is determined in various ways [34] and membrane voltage is measured in two compartment cells [35]. In order to know the effect of divalent ions, it is therefore logical to perform the same measurements with solutions that also contain divalent ions. Scientific literature with such measurements is limited to the publications of Kuno et al. [16], Oh et al. [15], Gómez-Coma et al. [36], and Avci et al. [37]. Apart from these, several publications report investigations into the effect of divalent ions in a complete RED stack [10,12,14,15,16,18,27,32,37]. The disadvantage of the latter method is that measured effects cannot be attributed in a direct way to the individual membranes. Moreover, stack resistance is the sum of ohmic, non-ohmic, and boundary layer resistance [38] and these values are influenced by spacer shielding and flow rates [39].

Conclusion. The negative influence of divalent ions on RED power is mainly caused by effects within the membranes and research should be focused on membrane resistance and membrane potential. Measurements of stack power gives additional information over boundary effects, uphill transport and non-ohmic resistance.

### 3.4. Measuring the Reduced Stack Voltage

The normal way to investigate the influence of divalent ions on RED power is starting with blank solutions that only contain the ions Na^+^ and Cl^−^ with concentrations of e.g., 1 and 30 g/L NaCl. Introducing divalent ions presents certain practical challenges. Addition of MgCl_2_ or Na_2_SO_4_ results in a different [Na^+^]/[Cl^−^] ratio and a change in the membrane voltage is the result of two effects (divalent effect and the ratio effect). The same holds for the resistance and power density of the system. Even in the case of addition of MgSO_4_—with no change of the [Na^+^]/[Cl^−^] ratio—the resistance of the feed water in the compartments between the membranes decreases and affects the power density. Moreover, the addition of extra ions—especially when they are divalent—causes an increase in the ionic strength, which in turn affects the activity of already present ions and consequently also on the membrane potential.

Instead of adding divalent ions, a better option is to substitute monovalent ions for divalent ions. This can be done in two ways: based on charge equivalence (i.e., normality = equivalents per liter) or on molarity (i.e., concentration = moles per liter). On charge equivalence, 1 mole NaCl is replaced by ½ mol MgCl_2_, Na_2_SO_4_ or MgSO4 and on molarity each mole NaCl is replaced by 1 mole divalent salt. Other parameters that can be kept constant in a series of salt mixtures are the conductivity and the ionic strength.

**Figure 4:**
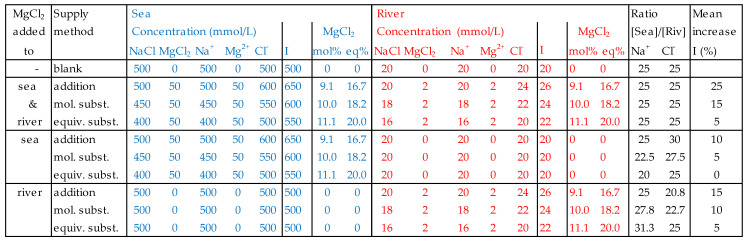
Experimental design for the determination of the effect of Mg^2+^ on the power of a RED stack. I = ionic strength. In the most right column, the increase of the ionic strength in the sea and the river compartment are averaged.

The consequences can be illustrated by the next example. Given a RED stack equipped with ideal membranes (i.e., permselectivities of 100%) and fed with pure NaCl solutions with concentrations of 500 mmol/L and 20 mmol/L, I aimed to investigate the effect of Mg^2+^ on the power of this RED stack. Table 1 shows that the Mg^2+^ content in seawater and in Lake IJssel water is about 10 mol%. Figure 4 shows the new concentrations after introduction of a similar amount of MgCl_2_ to the pure NaCl solutions according to the different methods (addition, molecular substitution, and equivalent substitution). In the rightmost columns, the concentration ratios of Na^+^ and Cl^−^ in sea and river water and the mean increase of the ionic strength in both feed waters are listed.

A good assessment of the effect of multivalent ions on the membrane resistance requires a background that is as consistent as possible. Changes in the concentration ratio of ions in sea and river water lead to changes in membrane voltages; changes in ion strength induce changes in activity coefficients and, therefore, also in voltages and conductivity. Introduction of MgCl_2_ in sea and river water (S + R) does not affect the concentration ratios. However, with equivalent substitution, the increase of ionic strength is the lowest and, therefore, this method is preferable here. Introduction of MgCl_2_ in only sea (S) or only river water (R) affects the concentration ratios of Na^+^ and Cl^−^ in all addition methods in a similar way. However, in both cases the increase in ionic strength is again the lowest with equivalent substitution. Therefore, equivalent substitution is the best option for these kinds of experiments.

Many authors who have investigated the effect of divalent ions on RED stacks have worked with divalent salt substitution on mole base. This makes interpretation of the intrinsic effect of the divalent ions difficult. However, it is possible to isolate the pure divalent effects on the OCV from molar substitution-based experiments. With MgCl_2_ for example, this is done as follows: (i) With the given OCV at 0% substitution, the mean permselectivity (*α*) is calculated using Equation (1). (ii) For each molar substitution with *p* mol% MgCl_2_, first the ionic concentrations are calculated if each mole of NaCl was changed for one mole imaginary ‘Na_2_Cl_2_‘, that is in practice, the addition of 2*p* mol% NaCl. The OCV of this cell pair with the new ionic concentrations is calculated with the known mean permselectivity. (iii) The measured OCV of the solution containing *p* mol% Mg^2+^ deviates from the hypothetical solution with 2*p* mol% Na^+^. This is represented by the OVC-factor:

(15) $$OCV_{factor}=\frac{\;\mathrm{measured}\;\mathrm{OCV}\;\mathrm{with}\;\mathrm{experimental}\;\mathrm{Mg}\;\mathrm{containing}\;\mathrm{solution}}{\mathrm{calculated}\;\mathrm{OCV}\;\mathrm{with}\;\mathrm{hypothetical}\;\mathrm{Na}\;\mathrm{containing}\;\mathrm{solution}\;}$$

The OCV-factor is plotted against the equivalent fraction of the divalent ions. The equivalent fraction *y* is derived from the molar fraction *x* as follows:

(16) $$y=\frac{2x}{x+1}$$

Useful data for further evaluation of the influence of divalent ions were found in the publications of Vermaas et al. [12], Avci et al. [37], Rijnaarts et al. [32], Kuno et al. [16], Oh et al. [15], Moreno et al. [14], and Pintossi et al. [27]. All these authors performed experiments in complete RED stacks, except Kuno et al., who measured the OCV on single membranes. The OCV was measured with different mixtures of NaCl and divalent salts (Na_2_SO_4_, MgCl_2_, CaCl_2_, or MgSO_4_). The results of the normalized measured voltages and corrected voltages differ significantly.

**Figure 5:**
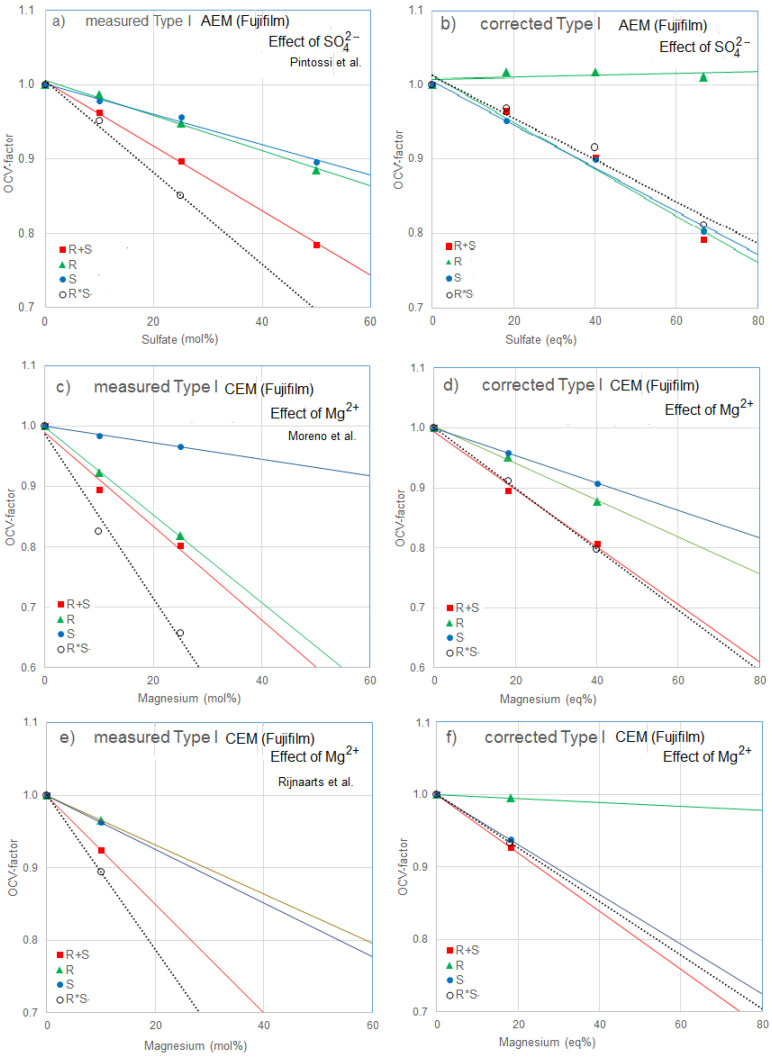
Effect of divalent ions on the OCV of cell pairs containing Type I AEM (**a**,**b**) and Type I CEM (**c**,**d**,**e**,**f**). Plots (**a**,**c,e**) were constructed from publications of Pintossi et al. [27], Moreno et al. [14], and Rijnaarts et al. [32]. By eliminating the effects induced by the changing monovalent ion concentrations, the corrected Figures (**b**,**d**,**f**) were constructed. In the legend, R refers to addition to river water, S to seawater, R+S to both feed waters and R*S to the calculated product of R and S.

An example with comparable membranes is given in Figure 5 where the effect of divalent ions is presented for three membranes Type I AEM (a and b) and Type I CEM (b, c, d, and e). These membranes are produced by Fujifilm (The Netherlands). Shown are the measured voltages (a, c, and d) and the corrected values (b, d, and e). The original voltages are plotted against the composition in mol% whereas for the corrected values are plotted against the equivalent percentage. Figure 5a was extracted from Figure 2 in the paper of Pintossi et al. [27]. Plots are shown where sulfate was added to both river and seawater (R + S), to river water only (R), and to seawater only (S). Moreover, I added the product of R and S (R*S). To all series, regression lines were added. Figure 5b was constructed after correction for the attribution of monovalent ions. The most intriguing conclusion is that interchanging two Na^+^ ions for one Mg^2+^ ion has no effect on the OCV if added to only the river water. Furthermore, because the R*S points coincidence almost with the R + S points in the corrected plots, there is no synergetic effect from R and S.

The influence of magnesium ions on several CEMs was studied by Moreno at al. [14] and Rijnaarts et al. [32]. Both research groups applied the same AEM (Fujifilm Type I) in combination with the different CEMs. Figure 5c is extracted from Figure 3 from the publication of Moreno et al. and Figure 5e with the aid of Table 1 from Rijnaarts at al. Figure 5d,f are the corrected versions of these. The similarity between Figure 5b,f is striking: the effect of magnesium is very similar to that of sulfate. Unfortunately, only measurements at 0 and 10 mol% were available.

However, despite the fact that Figure 5d,f relates to the same membrane pairs, the foregoing tendency is not visible at 3d. Differences between the experiments are the spacers used (Moreno et al.: 485 μm and Rijnaarts et al.: 200 μm).

**Figure 6:**
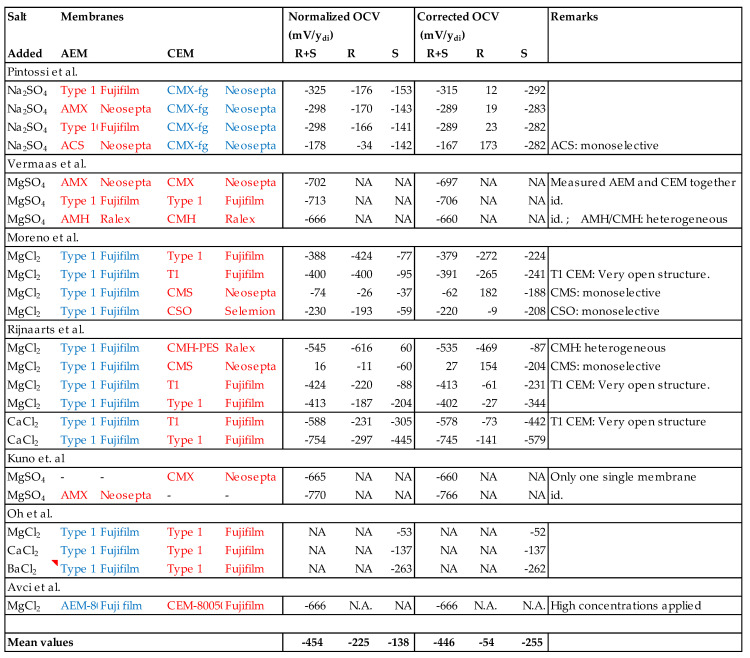
Results of the measured OCV as function of the divalent fraction before (normalized OCV) and after correction. Target membranes are shown in red and other membranes in blue. The slope of normalized and corrected values is given in millivolts per equivalent fraction of the divalent ions (mV/y_di_).

In the literature, 24 datasets describing the influence of divalent ions on the OVC of a RED stack were found. From all these datasets, regression lines were calculated for the normalized experimental OCV and the corrected OCV as function or the divalent equivalent fraction. Almost all research groups used total salt concentrations of 0.17 M together with 0.5 M (corresponding to 1 and 30 g NaCl/L). An exception is the work of Avci et al. [37] who used feed waters of 0.5 m together with 4 m. Figure 6 lists the regression lines of the OCV (in mV) as a function of the divalent equivalent fraction (y_di_). Most experiments were performed with 1:2 salts (Na_2_SO_4_, MgCl_2_, CaCl_2_, or BaCl_2_). In these cases, I assume that only one type of membrane in the stack is affected. Vermaas et al. applied MgSO_4_ in their experiments: in this case, the divalent influence the performance of both membrane types. Kuno et al. also used MgSO_4_ but their experiments were performed with only one membrane and the interpretation is here simple because only the membrane is affected.

A thorough statistical analysis of the results in Figure 6 is difficult because there were few similar membranes and if they are similar, it is not clear if there are differences in lot numbers, age, and pre-treatment. Furthermore, the stacks of the various researchers differ in terms of the number of membranes, spacer thickness, membrane dimensions, and leakage currents. There were also operational differences in flow rate and temperature. Therefore, only the mean values in the bottom row of the table in Figure 6 were initially considered for the conclusions.

- The mean values in the R and S columns of the corrected values show that multivalent ions have a much larger negative influence on the OVC when in seawater (mean = −255 mV/y_di_) than in river water (−54 V/y_di_). This is remarkable because with a high concentration of divalent ions in river water, a decrease of the OCV due to uphill transport is expected.
- Correction has little influence on the experiments where divalent salts were added in both feed waters.
- Addition of divalent ions to both feed waters has a larger effect than the sum of the separate effects (of addition to seawater and to river water).

### 3.5. Donnan Exclusion in NaCl Solutions Containing Divalent Ions

In most cases, the following specifications are given by the supplier of ion exchange membranes: ion exchange capacity (*IEC*), permselectivity (*PS*), area resistance (*R_a_*), swelling degree (*SD*), and thickness (*d*). Typical values for a Neosepta CMX membrane are: *IEC* = 1.62 equivalent/kg dry membrane, *PS* = 99.0%, *R_a_* = 2.91 Ω cm^2^, *SD* = 18%, *d* = 164 µm. The density of the dry membrane (*ρ*) is not reported generally but can be estimated to about 1000 g/L.

An important membrane property is the charge density (*CD*), the concentration of the fixed charges in the gel phase. It is obtained from the swelling degree (*SD*), density (*ρ*), and the ion exchange capacity (*IEC*). If it is assumed that the gel phase is created by the uptake of the water during swelling, then

(17) $$CD=\frac{IEC}{SD}\rho$$

For a CMX membrane—using *ρ* = 1000 g/L—it follows that CD = 9 mol/L.

**Figure 7:**
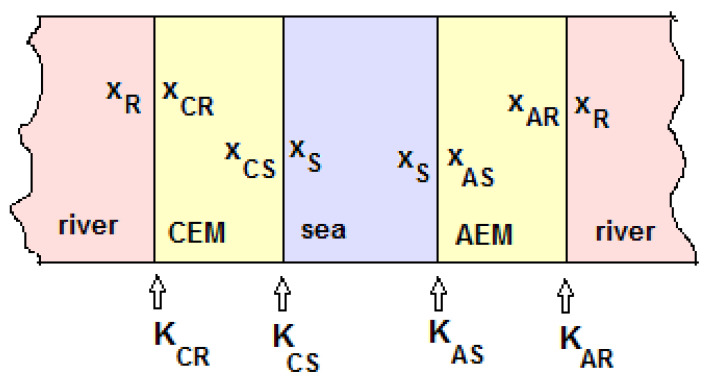
Mole fractions at the four interfaces.

On a CEM between two solutions called ‘sea’ and ‘river’, there are two interfaces: Sea−CEM and CEM−River. The molar fractions (*x*) in the water phase are indicated by the subscripts *S* (sea) and *R* (river). In the CEM phase the subscripts are *CS* (adjacent to the sea compartment) and *CR* (adjacent to the river compartment); in the AEM these are AS and AR. Figure 7 shows this convention.

On each interface, the mole fraction of the ions in the membrane phase are calculated by applying the theory of the Donnan equilibrium [40]. In this expression the power *z_i_* stands for the charge number of ion *i* (−2, −1, 1 or 2):

(18) $$\frac{x_{CR,i}}{x_{R,i}}={\left(K_{CR}\right)}^{z_{i}}\;\;\left(a\right);\;\;\frac{x_{CS,i}}{x_{S,i}}={\left(K_{CS}\right)}^{z_{i}}\;\;\left(b\right);\;\;\;\;\frac{x_{AS,i}}{x_{S,i}}={\left(K_{AS}\right)}^{z_{i}}\;\;\left(c\right);\;\;\frac{x_{AR,i}}{x_{R,i}}={\left(K_{AR}\right)}^{z_{i}}\;\;\;\left(d\right)\;\;\;$$

If concentrations are not too high, concentrations can be used instead of molar fractions. This leads to the next expression:

(19) $$\frac{C_{CR,i}}{C_{R,i}}={\left(K_{CR}\right)}^{z_{i}}\;\mathrm{etc}.$$

For a system with *n* different ions this gives *n* expressions. Because there are (*n* + 1*)* unknowns (the *n* concentrations and the equilibrium constant *K_CS_*), one more equation is needed. This is the electroneutrality equation (the fixed ions are also part of these):

(20) $$\;\sum_{i}z_{i}C_{CR,i}=0\;\;\mathrm{and}\;\mathrm{so}\;\mathrm{on}$$

**Figure 8:**
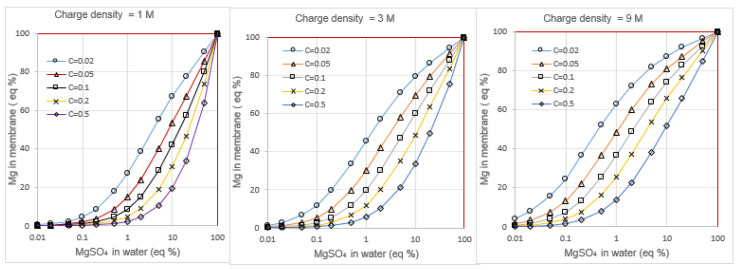
Calculated equivalent fraction of magnesium ions in cation exchange membranes as a function of the composition of the bulk water containing NaCl and MgSO_4_; the membranes have charge densities if 1 mol/L (**left**), 3 mol/L (**middle**), and 9 mol/L (**right**). The different plots in each figure represent different bulk concentrations.

Instead of molarity (molar concentration) C (in mol/L) of a specific ion, I prefer using normality N (in Eq/L). For monovalent ions, C and N have the same value. In the case of a divalent ion, the relationship is given by N = 2*C*. In the following, mainly solutions of NaCl with variable MgSO_4_ concentrations are considered. I apply the Donnan and electroneutrality equations to the membrane interfaces of CEMs with different charge densities and calculate the membrane concentrations of the four ions**.** Figure 8 shows the equivalent percentage of magnesium (Mg%) in the gel phase of three membranes (with charge densities of 1, 3, and 9 Eq/L) as function of the bulk Mg%. In Table 3, some data are presented from the ionic concentrations as found in sea and Lake IJssel feed water with total salt concentrations 0.006 Eq/L and 0.6 Eq/L. Magnesium and calcium ions are considered as indistinguishable because the Donnan exclusion model does not discriminate between Mg^2+^ and Ca^2+^. The same method is used for the prediction of the amount of sulfate in the outer sides of an AEM.

Consequently, the divalent cation equivalent fraction on the river water side within each considered CEM exceeds 95 eq% and, on the sea water side, these fractions are about 30% in the equilibrium state. In an open (not connected to a load) RED-stack with co-flow water supply, this is the situation at the entrance of the flow channel. During operation of the RED stack, the situation is difficult to predict. There is no equilibrium along the flow channel and the concentrations of both feed waters change. In this example with relatively high divalent cation concentrations, these ions move uphill (from low to high salinity) and spread across the membrane. The consequence is that the remaining places for Na^+^ are very limited, resulting in a greatly reduced Na^+^ transport from high to low concentration. Such reasoning also applies to the anions in an AEM. Applying membranes with lower charge density can reduce these effects somewhat, however, at the cost of a reduced exclusion of co-ions and thus probably with a negative effect on RED power. Charge densities of most commercial membranes are in the range 3–10 Eq/L and were tabulated by Tufa et al. [41].

A method to prevent the penetration of divalent ions into the membrane is the use of monovalent selective membranes. The exclusion of multivalent ions is based mainly on steric and/or Coulombic effects. For use in RED stacks, special monovalent selective membranes were prepared by Güler et al. [42]. Steric exclusion occurs due to a high level of cross-linking. An example is the ACS membrane from Neosepta [43]. Such a membrane is also less permeable to monovalent ions and it is questionable whether the application yields a net gain for the RED performance.

The second method is to apply a coating with an opposite fixed charge to that of the membrane. An example is the CSO membrane from Selemion, where a thin layer of polyethylene imine was applied to one side of this CEM. Sometimes both sides were covered with a charged layer, e.g., the CMS from Neosepta [10,28,43] (Some authors erroneously claim that this membrane derives its mono-selective properties from a highly crosslinked coating [14,24]). A recent development is the layer-by-layer method: instead of a single coating with a polyelectrolyte, various extremely thin layers are applied [24,28]. Coating with polyelectrolytes certainly limits the transport of divalent ions, but the membrane is still accessible for divalent ions, which again take up most of the space in the membrane and thus prevent the passage of Na^+^. Even if two-sided coating is carried out, divalent ions will still gain (slower) access to the membrane structure and will accumulate there until the Donnan equilibrium is reached. It is, therefore, questionable to what extent mono-selective membranes can contribute to a higher power density of a RED stack.

Conclusion. Monoselectivity is offset with a higher resistance and the benefits for RED are still debatable. A totally different way is to use very open membranes such as the T1-CEM and the T1-AEM from Fujifilm, which have a very low resistance. Any uphill transport of multivalent ions will certainly take place, but due to the open structure, the return transport of multivalent ions will take place quickly and an equilibrium will soon be established. Uphill transport takes place in the first part of the flow channel and after that the multivalent ions no longer interfere. Many researchers have, therefore, used these membranes in their RED stacks.

### 3.6. Formation of Ion Pairs in the Feed Water

Ion pairs are formed by association of an anion and a cation. They are common in aqueous solutions especially when multivalent ions are involved [44,45]. The ion pair may be an ion or a neutral particle. An example is [46]:

Mg^2+^ + SO_4_^2−^ ⬄ MgSO_4_ log(K) = 2.23

**Figure 9:**
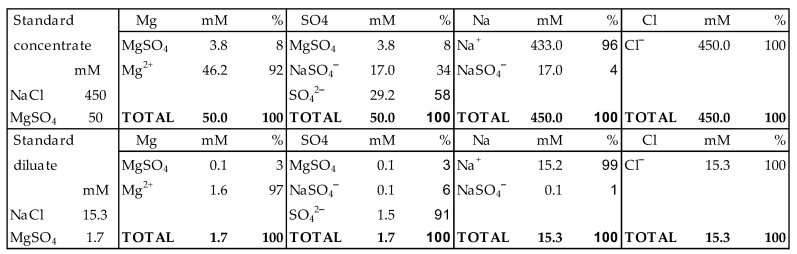
Ion pairs in standard concentrate and standard dilute according to OLI [47]. Only compounds are listed with more than 1% relative concentration.

Figure 9 lists the effect of ion pairing for NaCl-MgSO_4_ solutions of 0.5 M and 0.017 M, each containing 10 mol% MgSO_4_ and the question is to what extent does this ion pairing influence the RED process.

In membranes, the matter is more complicated than in solutions. Ion pairing can occur in the solution phase and in the gel phase. It is reasonable to assume that this process in the solution phase is identical to that in the bulk solution. In the gel phase, the concentration of co-ions is very low and only ion pairing between solved ions and fixed ions is expected. Consequently, transport of the ion pairs as listed in Figure 9 through an ion exchange membrane is unlikely because these pairs dissociate in the gel phase due to the very low concentration of the co-ions.

A theoretical study of ion pairing in cation exchange membranes, based on the Poisson–Nernst–Planck (PNP) equations, was performed by Magnifico [48]. He concluded that association between fixed and solved ions introduces a decreased membrane conductivity. Moreover, because in such a way, a part of the fixed charges is neutralized, also a decrease of the permselectivity is expected. Soldatov et al. [49] and Shaposhnik and Butyrskaya [50] performed ab initio calculations on the structure around a sulfonate group and an adjacent sodium ion. These data show that both ions are connected to each other via water molecules by means of hydrogen bonds. Badessa et al. used quantum mechanical calculations to elucidate the structure around mono- and multivalent ions as Na^+^, Ca^2+^, and Al^3+^ near a fixed sulfonate group [51,52]. They were also able to quantify the type of bonding between the counter ion and the fixed charge. With monovalent counter ions, the activation energy is mainly attributed to hydrogen bonds; with divalent ions, the energy of the ionic bond is half of the energy of the hydrogen bond, whereas with trivalent ions, the energy of the ionic and hydrogen bonds is comparable. However, such studies give a static view of the membrane structure and for ion transport the values of the formation constants are indispensable. With strong interaction between the ions, ion pairing will be an impediment to ion transport; however, at a less strong interaction, the pairing mechanism can even promote ion transport. This is the basis of the theory in which ions jump from one fixed charge to another. This concept was developed by Pourcelly et al. [53], Farhat et al. [54], and Yoroslavtev et al. [55,56]. Badessa et al. used the hopping model to explain why the mobility of double charged ions is lower than that of single charged ions [51,57]. Double charged ions, such as Ca^2^+, are connected to two fixed charges and each step involves that a single bond is broken and re-established with another fixed charge. During this process, the other bond remains intact, which means that the distance between the fixed charges should not be too great for good transport. This contrasts with the transport of singly charged ions, in which a bond has to be broken for each step, but after which the ion can freely search for a next fixed charge.

### 3.7. Gross Power Density

For commercial implementation of the RED process, the value of the maximum gross power density is a good indicator for the performance under real circumstances. Gross power density is the generated electrical power per m^2^ of applied membrane (CEM and AEM together). To avoid complications with asymmetric addition as described in Section 3.4, I searched scientific literature for experiments in which equal percentages of divalent ions were present in both feed waters. In all found publications, molar substitution was used, expressed in DMP (divalent molar percentage). It is true that the electrical conductivity of the feed waters increases with molar substitution, in contrast to equivalent substitutes, but the latter experiments are not available. Therefore, I obtained the power reduction percentage (*PRP*) from the publications with molar substitution experiments. From these parameters the relative divalent effect (*RDE*) was derived.

(21) $$RDE=\frac{PRP}{DMP}$$

**Figure 10:**
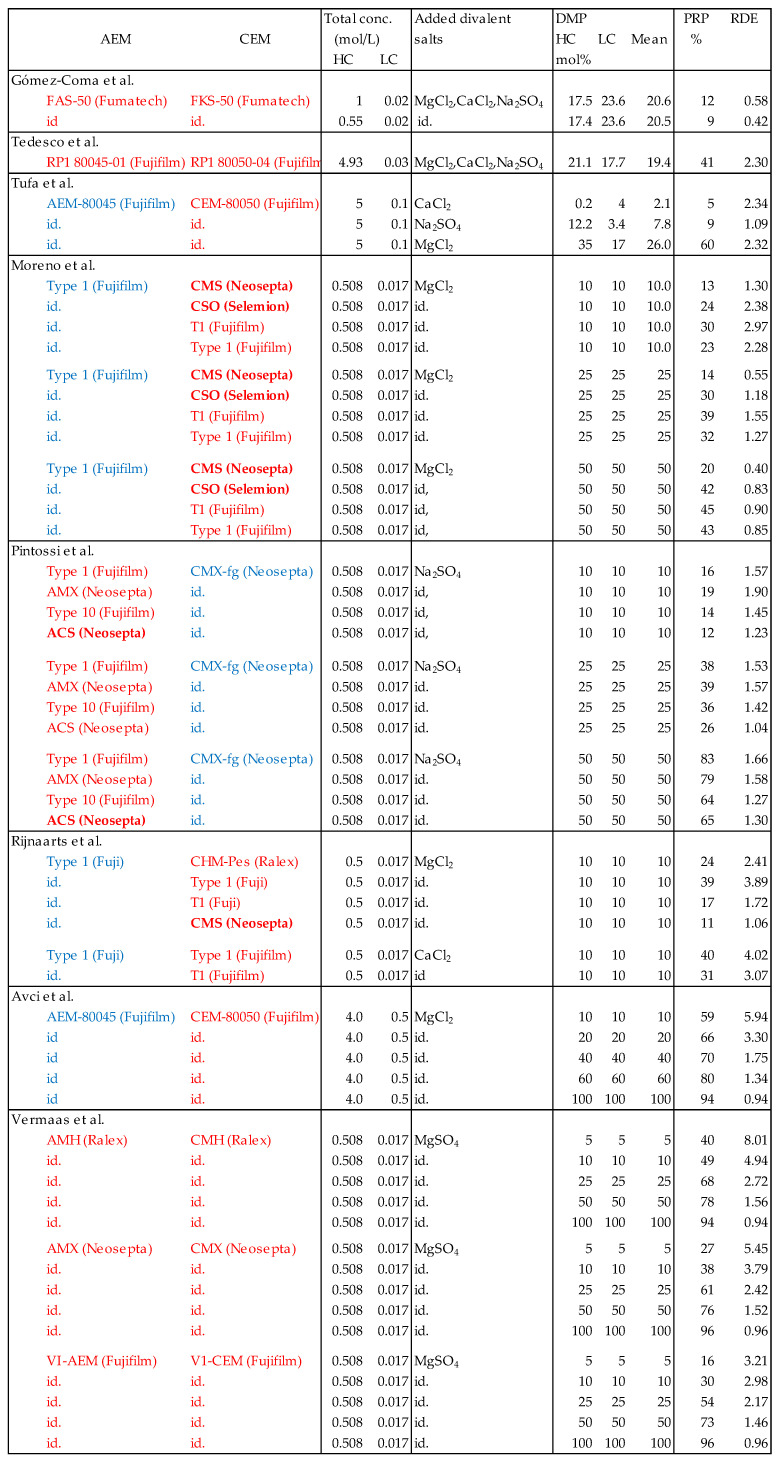
Power reduction percentage (*PRP*) and relative divalent effect (*RDE*) of divalent ions in different membranes. Target membranes are shown in red and auxiliary membranes in blue text. Monovalent selective membranes are shown in bold letter type.

**Figure 11:**
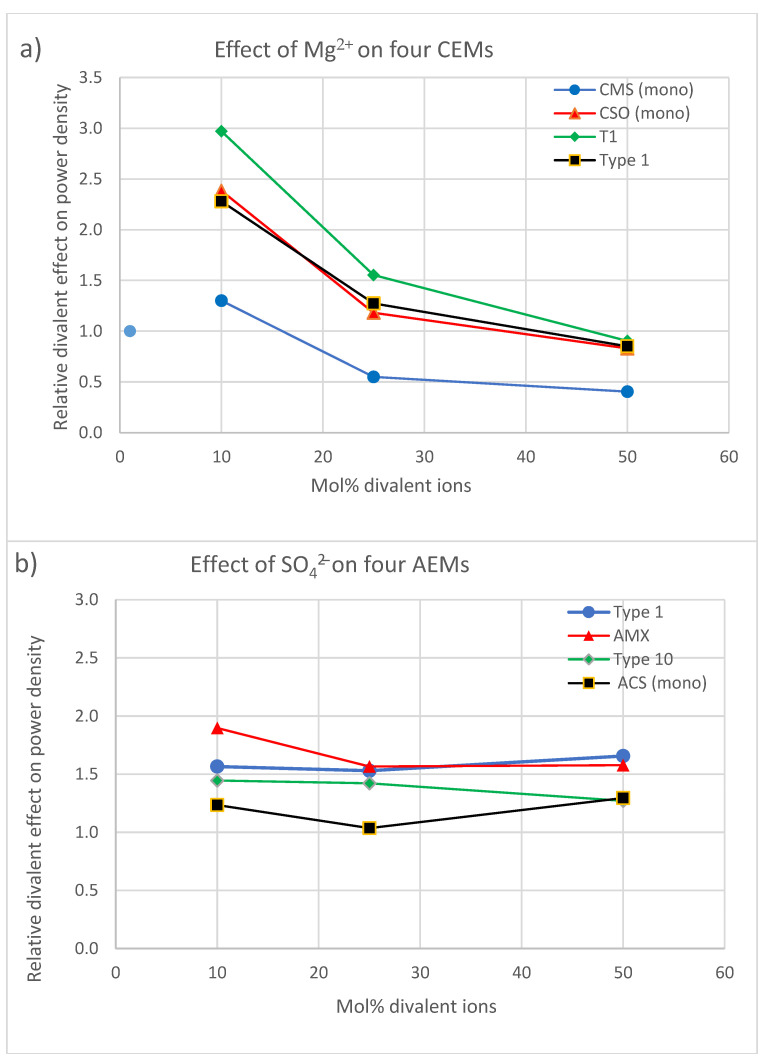
Relative divalent effect on power density for four CEMs (**a**) and four AEMs (**b**).

Figure 10 shows the resulting *RDE* values. In Figure 11, plots are constructed from the experiments of Moreno et al. [14] for the effect of Mg^2+^ on four different CEMs and from the experiments of Pintossi et al. [27] for the effect of SO_4_^2−^ on four AEMs. The following conclusions can be drawn:

- At lower concentrations (10 mol% divalent ions), the effect of Mg^2+^ on a CEM is generally higher than the effect of SO_4_^2−^ on an AEM; however, at high concentrations (50 mol% divalent ions), these effects are reversed.
- The membranes that suffer the least for both the CEMs and the AEMs are mono-selective membranes (CMS and ACS). However, from the CEMs, the CSO performs the same as the normal membranes. The monoselectivity of the CMS is based on a double-sided coating with a charged polymer, the CSO has such a charged layer only at one side and the ACS has on both sides a high cross-linked coating. In line with this, the question can be asked whether the CMS and ACS also continue to perform better during an endurance test or whether these membranes also become saturated with divalent ions after some time and therefore lose their unique properties.

### 3.8. Power Density and Efficiency

The exergy flow rate *X* of the feed waters to a RED stack is the Gibbs free energy per unit of time *t* [39]:

(22) $$X=\frac{\Delta G}{t}=2RT\left[\Phi_{R}C_{R}ln\frac{C_{R}}{C_{M}}+\Phi_{S}C_{S}ln\frac{C_{S}}{C_{M}}\right]\;\;\;\;\;with\;\;\;\;\;C_{M}=\frac{\Phi_{R}C_{R}+\Phi_{S}C_{S}}{\Phi_{R}+\Phi_{S}}$$

where *R* is the gas constant (*R* = 8.3145 J∙mol^−1^K^−1^), *T* the temperature (K) and Φ*_R_*, and Φ*_S_* the flow rates of river and sea water (m^3^/s). For equal flow rates of 1 m^3^/s and concentrations of *C_R_* = 17 and *C_S_* = 513 mol/m^3^ (i.e., 1 and 30 g/L), this amount is (at 25 °C) 1.79∙10^6^ W. Therefore, the power potential of the Rhine River with an average discharge of 2200 m^3^/s is almost 4 GW.

A quality parameter for stack performance is the power density *Pd*, the delivered electrical power *P* per *A* m*^2^* total membrane:

(23) $$Pd=\frac{P}{A}$$

Under real circumstances the generated power is less due to imperfect membranes, parasitic currents in the stack, non-equilibrium conditions, and the effects of multivalent ions. The ratio between power density *Pd* and the input exergy flow rate *X* is the energy efficiency *Y:*

(24) $$Y=\frac{Pd}{X}$$

There is a trade-off between efficiency and power density. For system optimization it can be useful to have a single target parameter that considers both power density and efficiency. The response parameter *R_P_* can be used for this purpose [58]:

(25) $$R_{P}=Pd\cdot Y$$

**Figure 12:**
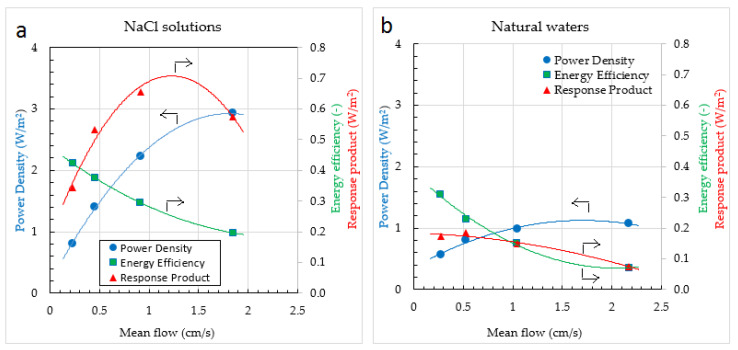
Power density, energy efficiency, and response product of a RED stack, fed with pure NaCl solutions (**a**) and fed with water from the Wadden Sea and the Lake IJssel (**b**). The stack consists of 10 cell pairs and is equipped with experimental membranes (Fujifilm, The Netherland). Stack dimensions are 10 × 10 cm^2^ and spacers are 115 μm. Parabolic regression lines are added to the plots. Arrows in the figures refer to the corresponding axes.

The RED pilot plant of the REDstack company on the Afsluitdijk in the Netherlands is developing the RED technique for economical use [20]. Improvements are made on pretreatment of the feed waters, stack design, membrane development, and power conversion to the electrical grid. Especially the effect of divalent ions is a point of special attention. Figure 12a shows the performance of a RED stack fed with pure NaCl solutions and Figure 12b the same stack fed with water from the Wadden Sea and the Lake IJssel. The differences are mainly due to the effect of divalent ions but there are also differences in temperature and concentrations. It is remarkable that there are large differences in power density (*Pd*) but the energy efficiency (*Y*) hardly differs.

**Figure 13:**
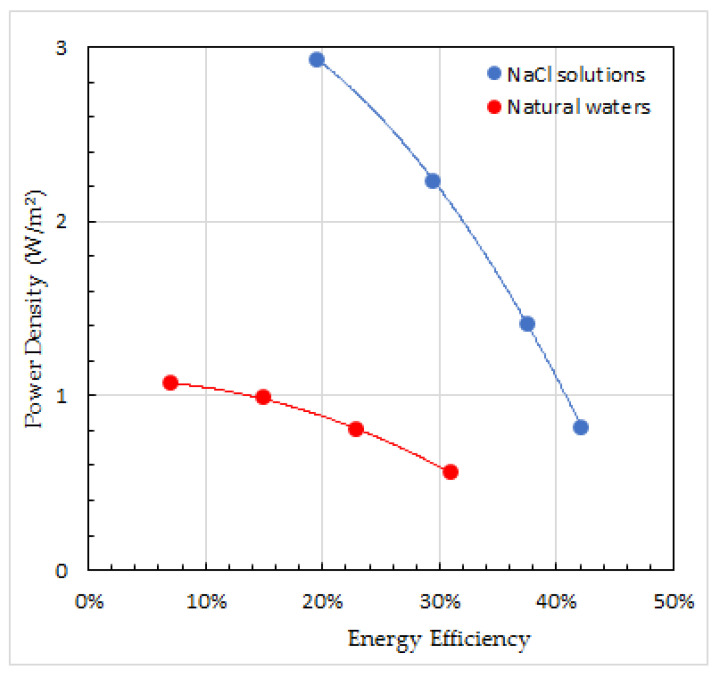
Relationship between power density and energy efficiency. The same RED stack is fed with pure NaCl solutions (blue line) and with water from the Wadden Sea and the Lake IJssel (red line). Parabolic regression lines are added to the plots.

A direct insight into the trade-off between power density (*Pd*) and energy efficiency (*Y*) is presented in Figure 13. The data used are the same as used in Figure 12. These plots are rather academic. For real applications, *Pd* and *Y* will also have to be corrected for the energy loss from the pressure drop. This aspect is discussed in earlier publications [58,59,60].

## 4. Conclusions and Perspectives

Divalent ions have a large negative influence on the RED process; they affect both power and efficiency. A lot of experimental work has therefore been conducted by various research groups with the aim of measuring the influence of these divalent ions. However, in several cases, incorrect assumptions have been made and conclusions drawn too quickly. The main conclusions are:

- The determining factor of whether uphill transport will occur is not the OCV but the membrane potential under power-producing conditions.
- In experiments investigating the influence of divalent ions on the OCV, the fact is often ignored that the addition of divalent salts also influences the concentrations of Na^+^ and Cl^−^ and, therefore, also the membrane voltage.
- The question is whether the apparent advantages of applying monospecific membranes in a RED stack will hold up during endurance tests.
- The effect of magnesium ions on CEMs is strongly concentration dependent in contrast to the effect of sulfate ions on AEMs.

Ion exchange membranes will be of great significance in the future. In addition to the role of these membranes in RED, there are also countless other applications (at least 23 listed in Bazinet and Georoy [61]). With ED, I think in the first place of desalination of seawater or brackish water, but ion-specific membranes are also increasingly used for the removal of unwanted ions or the isolation of wanted ions from various streams. In addition, IECs are used in Donnan dialysis and as separation sheets in electrolysis devices and redox flow cells.

Articles appear almost daily about the development of new membranes with claims regarding special properties such as specificity, mechanical strength, chemical resistance, durability, environmental friendliness, or production costs. However, for a real breakthrough in all above areas, it is necessary to develop test methods that make it possible to test these claims and to compare different membranes. In particular, the development of standard resistance measurements will remove a lot of uncertainty.

As to the theory of ion conduction, there remain great gaps in knowledge. There are various theories about the interaction between the counter ions and the fixed charges, which are mainly based on the transport of cations through CEMs; however, the process of anion transport by AEMs shows significant differences and the focus on these differences could contribute significantly to the theory.

I would therefore welcome an impetus to arrive at a universal test method for measuring the resistance of membranes in salt mixtures. Furthermore, agreement should also be reached on how a series of salt mixtures should be made in order to establish the relationships between the property to be measured and the addition.

## Figures and Tables

**Table 1:** Ionic composition of water from the Lake IJssel [21] and the North Sea [22].

	Lake IJssel mg/L mmol/L mol% meq/L eq%					Sea mg/L mmol/L mol% meq/L eq%				
Na^+^	41.4	1.8	46	1.8	30	10,556	459	86	459	78
K^+^	4	0.1	3	0.1	2	380	10	2	10	2
Ca^2+^	65	1.6	41	3.2	53	400	10	2	20	3
Mg^2+^	10.2	0.4	10	0.8	13	1262	52	10	104	18
total	120.6	3.9	100	6	100	12,598	531	100	592	100
HCO_3_^−^	141	2.3	35	2.3	32	140	2	0	2	0
Cl^−^	126	3.6	55	3.6	50	18,980	535	95	535	90
Br^−^	0.3	0	0	0	0	65	1	0	1	0
SO_4_^2−^	64.3	0.7	11	1.3	18	2649	28	5	55	9
total	331.8	6.5	100	7.2	100	21,836	565	100	593	100

**Table 2:** Molar ionic conductivities at infinite dilution at 298 K; data from [33].

Cations	λ_0_ S∙cm^2^ mol^−1^	Anions	λ_0_ S∙cm^2^ mol^−1^
Na^+^	50.11	Cl^−^	76.34
K^+^	73.52	Br^−^	78.4
½ Mg^2+^	53.06	I^−^	76.8
½ Ca^2+^	59.50	½ SO_4_^2−^	79.8

**Table 3:** Equivalent percentage of divalent ions in membranes with different charge densities (CD): CD = 1, CD = 32, and CD = 9 Eq/L as function of the total salt concentration and divalent fractions in the feed waters.

Feed Water	Salt Conc.(eq/L)	Divalent Ions in Feed (eq%)	Divalent Ions	Membrane	Divalent Ions in Gel Phase (Eq%)		
					CD = 1	CD = 3	CD = 9
Lake IJssel	0.006	68%	Mg^2+^/Ca^2+^	CEM	96%	98%	99%
Sea	0.6	21%	Mg^2+^/Ca^2+^	CEM	33%	48%	64%
Lake IJssel	0.006	18%	SO_4_^2−^	AEM	85%	92%	95%
Sea	0.6	9%	SO_4_^2−^	AEM	16%	29%	47%

## Data Availability

The data is available in this manuscript.

## Nomenclature

*Roman*	
A	ion diameter (pm)
a	activity (1)
C	concentration (mol/L)
CD	charge density (mol/L)
DMP	divalent molar percentage (%)
∆G	Gibbs free energy (J)
E	membrane voltage (V)
F	Faraday constant (96,485 C∙mol^−1^)
i	electrical current (A)
I	ionic strength (mol/L)
IEC	ion exchange capacity (eg/kg)
M	molarity (mol/L)
N	normality (Eq/L)
N	number of cell pairs
OCV	open circuit voltage (V)
P	power (W)
Pd	power density (Wm^–2^)
PRP	power reduction percentage (%)
R	gas constant (8.3145 J∙mol^−1^K^−1^)
RD	relative difference (1)
RDE	relative divalent effect (1)
Re	external resistance of a RED stack (load) (Ω)
Ri	internal resistance of a RED stack (Ω)
RP	response product (Wm^–2^)
P	power (W)
SD	swelling degree (1)
T	time (s)
T	temperature (K)
U	terminal voltage of a stack (V)
X	exergy flow rate (J/s)
Y	energy efficiency (1)
x	molar fraction (1)
y	equivalent fraction (1)
z	charge number (1)
*Greek*	
α	permselectivity (1)
γ	activity coefficient (1)
ρ	density (kg∙m^3^)
*Subscripts*	
m	monovalent
d	divalent
*Acronyms*	
AEM	anion exchange membrane
CEM	cation exchange membrane
DP	Donnan potential (V)
ED	electrodialysis
EMF	electromotive force
H	high concentration solution
L	low concentration solution
PNP	Poisson-Nernst-Planck
RED	reverse electrodialysis
SGE	salinity gradient energy
